# Complete F mitochondrial genomes of the Biwa pearl mussel, *Hyriopsis schlegelii*: the first report from the species’ native lake in Japan

**DOI:** 10.1080/23802359.2018.1534562

**Published:** 2018-10-26

**Authors:** Kohji Mabuchi, Kazuya Nishida, Nobuyoshi Nakajima

**Affiliations:** a Lake Biwa Branch Office, National Institute for Environmental Studies, Otsu, Japan;; b Environmental Genomics Office, National Institute for Environmental Studies, Tsukuba, Japan

**Keywords:** F mitogenome, *Hyriopsis schlegelii*, *Hyriopsis cumingii*, Lake Biwa, Illumina sequencing

## Abstract

We determined two complete mitochondrial sequences of female-transmitted (F) mitogenomes of two *Hyriopsis schlegelii* specimens from this species’ original habitat, Lake Biwa. The mitogenomes were both 15,951 bp in length, and the gene contents and orders agreed with those of the typical F mitogenome of *Hyriopsis*. Molecular phylogenetic analysis based on the previously identified 13 partial sequences confirmed that the two mitogenomes both belonged to *H*. *schlegelii* and not to a closely related Chinese species, *Hyriopsis cumingii.* The same analysis revealed that two mitogenomes (HQ641406 and FJ529186) previously published as *H*. *schlegelii* and *H*. *cumingii* might be misidentified.

*Hyriopsis schlegelii* is a freshwater pearl-producing bivalve originally endemic to Lake Biwa in central Japan. This species was known worldwide as the Biwa pearl mussel but is now threatened in its original habitat and classed as a critically endangered species (CR + EN) in the Japanese Red Data Book (Ministry of the Environment 2005). One probable threat to wild *H*. *schlegelii* populations is genetic contamination from the closely related Chinese species *Hyriopsis cumingii* (Matsuda 2016) although this theory has not yet been confirmed by a genetic survey. Genetic differences between the two species were clarified via partial sequencing of the COX2-COX1 region of mitochondrial (mt) DNA and partial ITS1 region sequencing of nuclear DNA by Shirai et al. (2010) in their molecular phylogenetic study, which primarily used 20 specimens collected from Lake Biwa before the introduction of *H*. *cumingii*. However, the whole mitochondrial genome (mitogenome) sequence of *H*. *schlegelii* in its native habitat has not previously been reported.

In this study, we determined the mitogenome sequences of two specimens of *Hyriopsis* mussel from a pearl culture farm in Lake Biwa. The specimens were deposited at Lake Biwa Museum, Shiga Prefecture, Japan, under registration numbers LBM-1300014556 and LBM-1300014557. Genomic DNA was isolated from the specimens’ foot muscle tissue and sequenced using an Illumina MiSeq (Illumina). The resultant reads were assembled using CLC Genomic Workbench (ver. 11.01; QIAGEN). Contigs were annotated by alignment with a female-transmitted (F) mitogenome sequence published as *H*. *cumingii* (FJ529186). Using the four available F mitogenomes of *Hyriopsis* (including the two sequenced here) and the F mtDNA sequences of *Hyriopsis* published in Shirai et al. (2010), we constructed a phylogenetic tree using a supermatrix approach (de Queiroz and Gatesy 2007); the method of tree construction is described in the legend of Figure 1.

**Figure 1.**
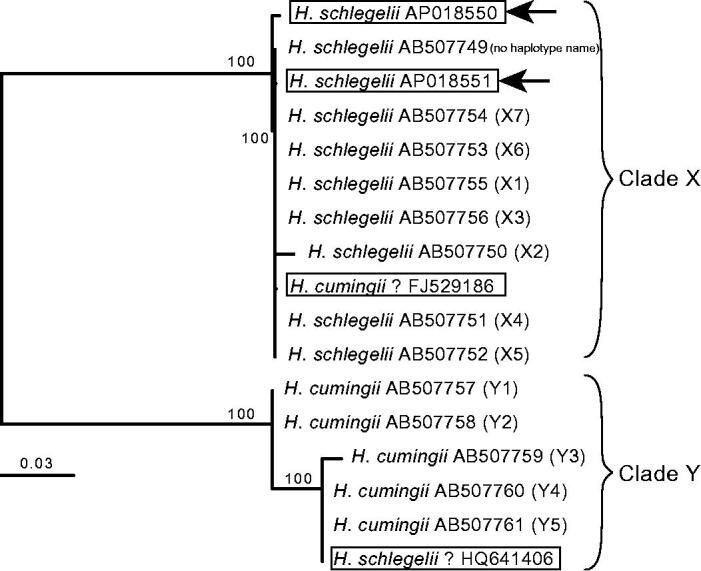
Supermatrix tree of four whole mitochondrial (15,939–15,954 bp) and 13 partial COX2-COX1 region (1027 bp) sequences of female-transmitted (F) mitogenomes of *Hyriopsis schlegelii* and *H*. *cumingii*. Bootstrap support (80% or over) is indicated at the nodes. Accession numbers are indicated after the species names with haplotype names of Shirai et al. (2010) in parentheses for partial sequences. Species names and accession numbers of the four mitogenomes are boxed, and the two mitogenomes sequenced in this study are indicated by arrows. The tree backbone was first generated for the four mitogenomes by the neighbor-joining (NJ) method using the online version of MAFFT (https://mafft.cbrc.jp/alignment/server/). The obtained NJ tree was then used as a backbone constraint for the supermatrix tree. The supermatrix tree was constructed based on the dataset including the four mitogenome and 13 partial sequences, which were first multiple aligned using MAFFT and corrected by eye using Mesquite (version 3.31; http://www.mesquiteproject.org). For the resultant 16,003-bp dataset, maximum likelihood analysis was performed using RAxML BlackBox (https://embnet.vital-it.ch/raxml-bb/).

The mitogenome sequences obtained (DDBJ accession nos. AP018550 and AP018551) were both 15,951 bp in length. Male (M) and female (F)-transmitted mitogenomes in freshwater mussels are known to have different gene orders (Breton et al. 2009). The gene order of our mitogenomes differed from that of the *H*. *cumingii* M mitogenome (KC150028: 17,100 bp) but was identical to this species’ F mitogenome (FJ529186: 15,954 bp), which confirmed that the newly obtained sequences were those of the F mitogenome.

The resultant supermatrix tree recovered two major clades of the tree published in Shirai et al. (2010): clade X corresponds to *H*. *schlegelii* and clade Y to *H*. *cumingii* (Figure 1). Our two mitogenomes were both nested within clade X, which confirmed they came from *H*. *schlegelii*. These two sequences are the first F mitogenome sequences of *H*. *schlegelii* reported from its native habitat. Based on their phylogenetic positions in the tree, however, the two remaining mitogenomes, FJ529186 (published as *H*. *cumingii*) and HQ641406 (published as *H*. *schlegelii*), seem to have been misidentified. These possibly misidentified sequences were reported from China, where the native *H*. *cumingii* has been hybridized with the introduced *H*. *schlegelii* for selective breeding (Peng et al. 2014).

## Geolocation information

35°05’40.34”N 135°56’51.01”E
